# Detection of RBC-bound antibodies using flow cytometry and their correlation with HLA polymorphism

**DOI:** 10.3389/fimmu.2026.1743789

**Published:** 2026-07-22

**Authors:** Anwar Ullah, Lina Liu, Xia Qi, Hui Liu

**Affiliations:** College of Medical Laboratory, Dalian Medical University, Dalian, China

**Keywords:** agglutination, flow cytometry, HLA polymorphism, red blood cells, sequence-based typing

## Abstract

**Background:**

The immune response in elderly individuals is influenced by genetic factors, particularly polymorphisms in major histocompatibility complex (MHC), such as human leukocyte antigen (HLA). Age-related changes in immune function, referred to as immunosenescence, can lead to the formation of RBC-bound antibodies, which may pose risks in transfusion medicine.

**Objective:**

This study aims to investigate the association between HLA-DQB1 alleles and the presence of RBC-bound antibodies in elderly individuals.

**Materials and methods:**

A total of 83 healthy elderly (aged 60-70) samples were collected. Blood samples were processed by centrifugation at 3500 rpm for 10 min to obtain serum. RBCs were isolated through centrifugation at 3000 rpm, washed with saline, and 2% type O RBC was prepared. Serum (100 µL) was mixed with saline (100 µL) and 2% type O RBCs (50 µL), incubated at 37 °C for 30 min, and analyzed via flow cytometry. At the same time, control sample was also processed with equal volume. DNA was extracted using the sequence-based typing kit, and specific genes at the HLA DQB1 locus were amplified by PCR, confirmed through agarose gel electrophoresis and sequencing for allele identification.

**Results:**

RBC-bound antibodies were detected in 42 of 83 participants, indicating a higher index of agglutination (IAG), while 41 samples showed no RBC-bound antibodies, indicating negative IAG. The mean IAG was *-1.88* (*SD = 25.943*), reflecting variability in immune reactions. Sequence based typing (SBT) analysis revealed a significant association between the HLA-DQB1*04 allele and IAG *(p < 0.034)*, while no significant associations were found for alleles DQB1*02, *03, *05, and *06.

**Conclusion:**

This study highlights the role of HLA-DQB1*04 in the presence of RBC-bound antibodies in elderly individuals, emphasizing the complexities of immunosenescence and variability in immune responses within this population.

## 1 Introduction

The human major histocompatibility complex (MHC) was discovered over 50 years ago, also referred to as HLA, and initially gained prominence for its role in transplantation and histocompatibility antigens. Extensive research has illuminated the complex genomic makeup of the MHC, revealing how genetic variance within this region significantly impacts susceptibility to autoimmune, infectious, and diverse diseases. With high gene density and polymorphism, the MHC presents a challenging interplay of genetic diversity with profound functional consequences. Despite the complexities of its diversity, significant insights have emerged, establishing the MHC as a foundational element in human genomics (1–3). The MHC in humans, located on chromosome 6, is a region of the genome pivotal for immune function and self-recognition. It encodes a diverse set of genes responsible for the production HLAs, which play a crucial role in antigen presentation to T cells. The MHC is divided into two main classes, Class I and Class II. Class I molecules, including HLA-A, HLA-B, and HLA-C, are expressed on nearly all nucleated cells and present intracellular antigens to cytotoxic T cells (4–6). MHC class II molecules hold a critical position within the immune system, playing a pivotal role in combating infections and serving as a primary concern in the field of transplantation medicine. These molecules not only present antigenic peptides sourced mainly from extracellular origins to CD4+ T cells but also facilitate the crucial process of thymic selection for helper T cells. Comprising an α and β chain, MHC class II molecules are guided to endosomal-lysosomal compartments by the invariant chain, ensuring their proper function in antigen presentation and immune response modulation. Class II molecules, such as HLA-DP, HLA-DQ, and HLA-DR, are primarily found on antigen-presenting cells and present extracellular antigens to helper T cells. The extreme polymorphism of HLA genes within the MHC is a hallmark of the complex, allowing for a vast array of antigen presentation capabilities crucial for immune surveillance and response (7–11).

The MHC significance extends beyond immune responses, playing a key role in various diseases, including autoimmune disorders, infectious diseases, and transplantation outcomes. The diversity of HLA alleles within the MHC poses challenges in transplantation matching, as mismatched HLA types can lead to graft rejection. In autoimmune diseases, associations with specific HLA alleles shed light on genetic predispositions and immune dysregulation. Furthermore, the MHC involvement in infectious diseases is highlighted by its role in shaping individual immune responses to pathogens. HLA class I and class II antigens exhibit remarkable polymorphism in their structural genes, making them one of the most diverse gene families in humans. This extensive genetic diversity results in minor variations in amino acids within each HLA molecule among individuals. Such variability gives rise to a wide array of unique HLA types, contributing to the complexity of the human immune system (12–15).

The HLA is an important component of the immune system, have a significant role in autoimmune diseases, there is often a dysregulation in the recognition of self from non-self-antigens, leading to an immune attack on the body’s own tissues. Specific HLA alleles have been associated with an increased risk of developing various autoimmune diseases. For example, certain HLA alleles are linked to autoimmune conditions such as type 1 diabetes, rheumatoid arthritis, and celiac disease. The interactions between HLA molecules and self-antigens can trigger an immune response against the body’s own tissues, contributing to the pathogenesis of autoimmune diseases (16–18).

Flow cytometry operates by analyzing and profiling individual cells or particles within a liquid suspension as they traverse a laser beam. This method quantifies various cellular traits like size, granularity, and fluorescence, delivering detailed cellular insights quickly and effectively. Detection of RBC-bound antibodies via agglutination using flow cytometry techniques stands as a sophisticated and precise tool in immunohematology (19–21). It offers high sensitivity and specificity in identifying and characterizing RBC-bound antibodies, vital for clinical applications such as blood transfusions and diagnosing autoimmune hemolytic anemia. By leveraging flow cytometry ability to analyze cells based on their physical and chemical features, researchers and clinicians can efficiently detect and measure RBC-bound antibodies with heightened precision and efficiency. This not only streamlines antibody detection but also yields valuable insights into the immune response to RBC antigens, enhancing patient care in transfusion medicine (22–24).

This study explores RBC-bound antibodies and their association with HLA polymorphism using flow cytometry and SBT techniques. To overcome the limitations of traditional serological methods in distinguishing subtle antigenic differences, DNA-based techniques were introduced to provide a higher level of typing resolution. This combined approach improves compatibility assessments and supports better management of alloimmunization risks in transfusion and hematology practices.

## 2 Materials and methods

### 2.1 Ethical statement

The research followed the principles outlined in the Declaration of Helsinki. Approval for the study was obtained from the Dalian Medical University Ethics Committee, considering that the samples utilized were residual from clinical procedures and not collected specifically for this study, thereby posing no risk to the patients.

### 2.2 Subjects

The study population consisted of 83 healthy Chinese elderly individuals. Blood samples were collected from the Dalian Blood Bank Center, maintaining an equal distribution of male and female participants within an age range of 60 to 70 years. Inclusion criteria were healthy status with no history of autoimmune diseases, malignancy, or recent blood transfusion. Exclusion criteria included acute infection, fever, or use of immunosuppressive medications. Approximately 2 mL of blood was collected into EDTA tubes for analysis.

#### 2.2.1 Serum preparation

Blood was drawn into plain tubes and stored at 4 °C for 1 hour, followed by centrifugation at 3500 rpm for 10 min to separate the serum. Serum samples were then heat-inactivated by placing them in a water bath at 56 °C for 30 min before being utilized for further experimental analysis.

#### 2.2.2 Preparation of 2% RBCs

The type O whole blood was collected into a anticoagulant tube, and centrifugation at 3000 rpm for 5 min to isolate the RBCs. Following centrifugation, the plasma was removed, and the residual RBCs were mixed with normal saline. After thorough mixing, the tube underwent another centrifugation at 3000 rpm for 5 min. This step was repeated twice to remove the supernatant and achieve a transparent liquid. To obtain 2% RBC solution, 20 µL of type O RBCs were combined with 980 µL of normal saline.

### 2.3 Reagents and instruments

Reagents and instruments used in the study included the whole blood from type O elderly healthy individuals, normal saline, flow Cytometer Agilent NovoCyte 2040R. a Thermal Cycler with a heated cover, adjustable pipettes, a Vortex mixer, disposable pipette tips, molecular biology grade agarose, 0.5x TAE or TBE buffer, ethidium bromide solution, a photographic or image documentation system, a hot plate or microwave oven for heating agarose solutions, molecular biology grade agarose, distilled water or DNA diluents, a UV transilluminator, and the Sequence based technique (SBT Kit) from TBG Biotechnology Corp (China).

### 2.4 Detection of RBC-bound antibodies in elderly individuals based on flow cytometry

Serum (100 µL) was taken from 83 elderly individual’s age (60-70), with an equal distribution of males and females. This serum was combined with an equal volume of normal saline (100 µL) containing 2% type O RBCs (50 µL) in eppendorf tubes. At the same time, control sample was also prepared by substituting serum with normal saline in the eppendorf tubes with equal volume. The tubes were then incubated in a water bath at 37 °C for 30 min. After incubation, one drop of anti-human globulin (AHG) was added to each tube and allowed to stand at room temperature for 5 min. All samples, including control, were thoroughly mixed and analyzed using flow cytometry. To ascertain the index of agglutination (IAG), the following parameters were configured for flow cytometry analysis: the FSC-H threshold was established at over 100,000, with a stop condition set at 20 µL. The sample flow diameter was measured at 7.2 μm, and the sample flow rate was fixed at 14µL/min. Subsequent to the analysis of all samples via flow cytometry, the Absolute count value for each sample was documented. To compare the values of the original and control group samples, IAG was used, based on the following equation:


$$IAG=\;\left(1-Observed\;sample\;÷\;Control\;samples\right)\;\times 100\%$$

On a scale of 0 to 1, index of agglutination reflects the intensity of agglutination, where a higher value signifies a stronger clumping phenomenon when RBCs meet antibodies. This quantitative measure allows for precise comparisons across different samples, ensuring reliable interpretation in clinical and laboratory settings. A value close to 1 indicates near-complete agglutination, suggesting a potent antibody-RBC interaction, while a value near 0 implies minimal or no clumping, typical of a negative reaction. The index serves as a critical tool in blood typing, antibody screening, and transfusion compatibility testing, where even slight variations can have significant diagnostic implications.

### 2.5 Detection of HLA haplotypes using sequence-based typing technique

In this study, the blood samples from elderly individuals were initially analyzed using flow cytometry to assess the IAG. Furthermore, to confirm the presence of different HLA alleles at the molecular level, we employed SBT technique. This approach was applied to identify the roles of various alleles and their association with RBC-bound antibodies. The HLAssure™ SE SBT Kit developed by (TBG Biotechnology Corp china) was used for the extraction and analysis of genomic DNA from whole blood samples. The extraction process followed a standard and reliable protocol to ensure high-quality DNA. After extraction, polymerase chain reaction (PCR) was conducted to amplify specific genes associated with the HLA DQB1 locus, utilizing sequence-specific primers included in the kit. The effectiveness of the amplification was confirmed through agarose gel electrophoresis, which allowed for the visualization and verification of the PCR products. Once the amplification was validated, the resulting DNA was purified and subjected to sequencing reactions to accurately identify the specific DQB1 alleles (*02, *03, *04,*05,*06) present in the samples. The sequences obtained were then compared to established reference sequences to ensure precise HLA typing, facilitating the detection of genetic polymorphisms.

Quality control for SBT included verification of DNA integrity by 1% agarose gel electrophoresis. A PCR negative control (no template) was included in each amplification run. Bidirectional sequencing was performed for all samples, and allele assignment was validated using HLA AccuType™ software against the established reference database.

### 2.6 Statistical analysis

The frequencies of HLA genotypes across various alleles were evaluated for deviations from Hardy-Weinberg equilibrium using R programming. Both expected and observed frequencies were compared utilizing a student’s t-test. The distribution of IAG was assessed through a single-sample test. Furthermore, the association between HLA alleles and IAG was statistically analyzed using the chi-square test, with significance defined as *p* values less than *0.05*. All data analyses were conducted using SPSS version 25 (IBM SPSS Statistics for Windows, Version 25).

## 3 Results

### 3.1 Genetic diversity and equilibrium in HLA-DQB1 allele distribution

The data summarized in **Table 1** presents the allele frequencies of HLA-DQB1*02, HLA-DQB1*03, HLA-DQB1*04, HLA-DQB1*05, and HLA-DQB1*06 among male, female, and total individuals within the studied population. The percentages reflect the proportion of each allele within the respective demographic groups. These allele frequencies provide a foundational basis for subsequent analyses aimed at comparing the observed and expected frequencies of allele combinations, thereby enhancing our understanding of the genetic composition and diversity present within the population.

**Table 1:** Distribution of HLA-DQB1 alleles by gender.

Allele	Male	Female	Total
HLA-DQB1*02	20 (25.0%)	13 (15.3%)	33 (19.87%)
HLA-DQB1*03	25 (31.3%)	36(42.4%)	61 (36.74%)
HLA-DQB1*04	5 (6.3%)	4 (4.7%)	9 (5.45%)
HLA-DQB1*05	7 (8.8%)	8 (9.4%)	15 (9.03%)
HLA-DQB1*06	23 (28.7%)	25(29.4%)	48 (28.91%)

**Figure 1:**
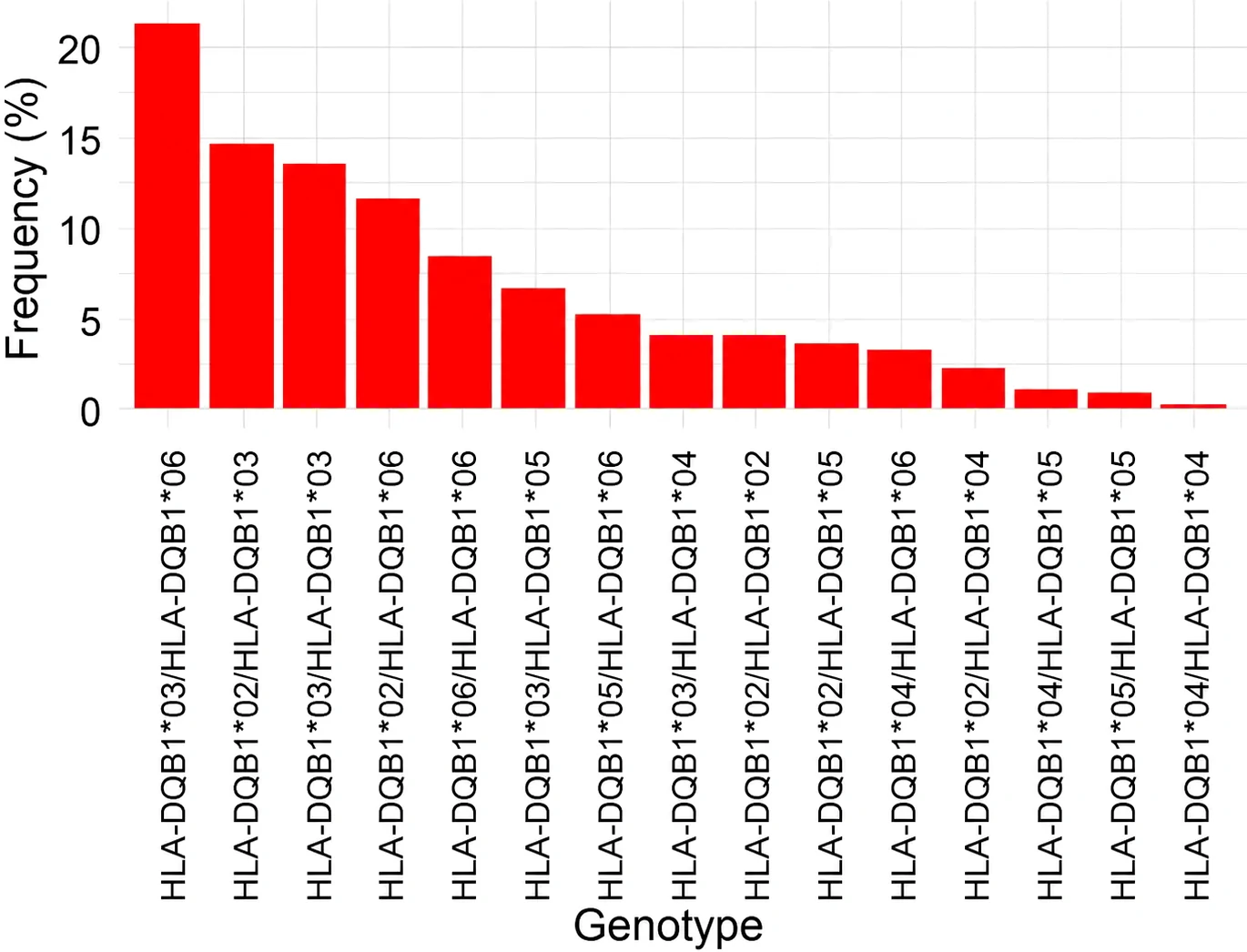
Hardy-Weinberg genotype frequencies. This figure shows the distribution of homozygous and heterozygous genotypes for the studied alleles, comparing observed frequencies with expected Hardy-Weinberg equilibrium values.

The expected and observed frequencies of specific homozygous and heterozygous allele combinations are listed, providing a detailed comparison between the predicted genotype frequencies under the Hardy-Weinberg equilibrium and the actual frequencies observed in the population. Notably, the *p*-values associated with each allele combination signify the level of agreement between the observed and expected frequencies. A high *p*-value like the one obtained (*0.976*) suggests that the population’s genetic makeup closely follows the Hardy-Weinberg equilibrium, indicating genetic stability and equilibrium within the studied population. These findings underscore the reliability of the genetic data obtained through sequence-based typing and the consistency of allele frequencies within the population, contributing significantly to our understanding of genetic dynamics and allele distribution patterns as shown in **Table 2** and **Figure 1**.

**Table 2:** Expected and observed frequencies according to Hardy-Weinberg Law.

Allele	Expected Frequency	Observed Frequency	*p-*value
HLA-DQB1*02/HLA-DQB1*03	0.1460	0.169	0.976
HLA-DQB1*02/HLA-DQB1*05	0.0359	0.036	
HLA-DQB1*02/HLA-DQB1*06	0.1149	0.096	
HLA-DQB1*02/HLA-DQB1*02	0.0395	0.036	
HLA-DQB1*03/HLA-DQB1*05	0.0664	0.084	
HLA-DQB1*03/HLA-DQB1*06	0.21**2**4	0.193	
HLA-DQB1*05/HLA-DQB1*06	0.0522	0.024	
HLA-DQB1*03/HLA-DQB1*03	0.1350	0.12	
HLA-DQB1*05/HLA-DQB1*05	0.0082	0.012	
HLA-DQB1*06/HLA-DQB1*06	0.0836	0.12	
HLA-DQB1*02/HLA-DQB1*04	0.0217	0.012	
HLA-DQB1*03/HLA-DQB1*04	0.0400	0.06	
HLA-DQB1*04/HLA-DQB1*05	0.0098	0.00	
HLA-DQB1*04/HLA-DQB1*06	0.0315	0.036	
HLA-DQB1*04/HLA-DQB1*04	0.0030	0.00	

### 3.2 Agglutination results in elderly individuals

**Figure 2:**
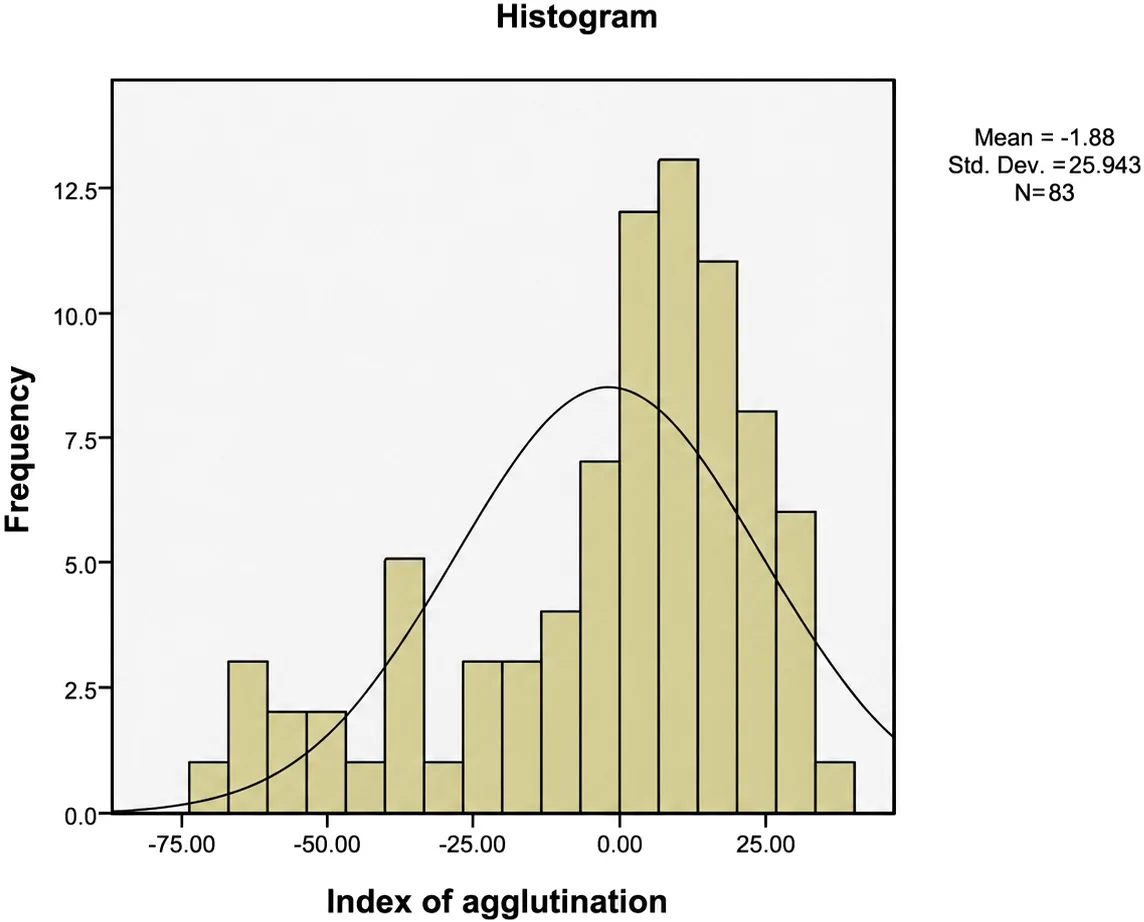
IAG frequencies in elderly individuals based on flow cytometry. This figure shows the distribution of IAG frequencies, with 42 out of 83 participants testing positive for RBC-bound antibodies and a mean IAG of -1.88.

In the study of elderly individuals, RBC-bound antibodies were detected in 42 out of 83 participants, indicating a positive IAG, while no RBC-bound antibodies were observed in the remaining 41 samples, reflecting a negative IAG. The mean IAG was calculated to be -1.88, with a standard deviation of 25.943. The positive agglutination results in 42 samples suggest that antibodies are binding to RBCs, indicating an immune response and the presence of specific antigens that are recognized by these antibodies. In contrast, the negative agglutination results in 41 samples imply the absence of RBC-bound antibodies, suggesting a lack of immune reactivity to the antigens tested. The calculated mean IAG of -1.88 provides an average measure of the agglutination responses observed across the sample set, while the standard deviation of 25.943 indicates the degree of variability or dispersion of the agglutination values around the mean. This variability in agglutination responses reflects the diversity of immune reactions and the range of antibody binding affinities present within the sample population. The results derived from the IAG underscore the distribution of positive and negative agglutination patterns among the tested samples, offering valuable insights into immune system behavior and antigen-antibody interactions within the context of this study, as illustrated in **Figure 2**.

### 3.3 HLA-DQB1 and RBC-bound antibody association results in elderly individuals using SBT

A total of 42 elderly individuals tested positive for IAG, while 41 individuals exhibited negative IAG results, determined through flow cytometry analysis, which assesses the presence of antibodies bound to red blood cells indicative of an immune response. To investigate the genetic factors associated with these immune responses, HLA DQB1 alleles were analyzed using the Sequence-Based Typing (SBT) technique, allowing for precise identification of specific haplotypes. Among the various haplotypes, the HLA-DQB1*04 allele showed a significant correlation with IAG, evidenced by a *P*-value of 0.034, suggesting its pivotal role in the development of RBC-bound antibodies that support the agglutination phenomenon. In contrast, other haplotypes (*02, *03, *05, 06) did not demonstrate significant associations with IAG, indicating they may not influence immune response in the same manner as shown in **Table 3**. These findings highlight the importance of the HLA-DQB1*04 allele in understanding immune responses in the elderly and suggest pathways for further research into the relationship between genetic factors and immune function.

**Table 3:** Association between HLA-DQB1 haplotypes and RBC-bound antibodies with respect to IAG.

HLA	Groups	Positive rate of Ab to RBC	*X* ^2^	*p-value*
DQB1*02	Carry	17 (51.5%)	0.014^a^	1.000
	Not- carry	67 (50.4%)		
DQB1*03	Carry	29 (46.8%)	0.580^a^	0.521
	Not- carry	55 (52.9%)		
DQB1*04	Carry	8 (88.9%)	5.580^a^	0.034
	Not- carry	76 (48.4%)		
DQB1*05	Carry	6 (40.0%)	0.742^a^	0.429
	Not- carry	78 (51.7%)		
DQB1*06	Carry	24 (50.0%)	0.010^a^	1.000
	Not- carry	60 (50.8%)		

**Figure 3:**
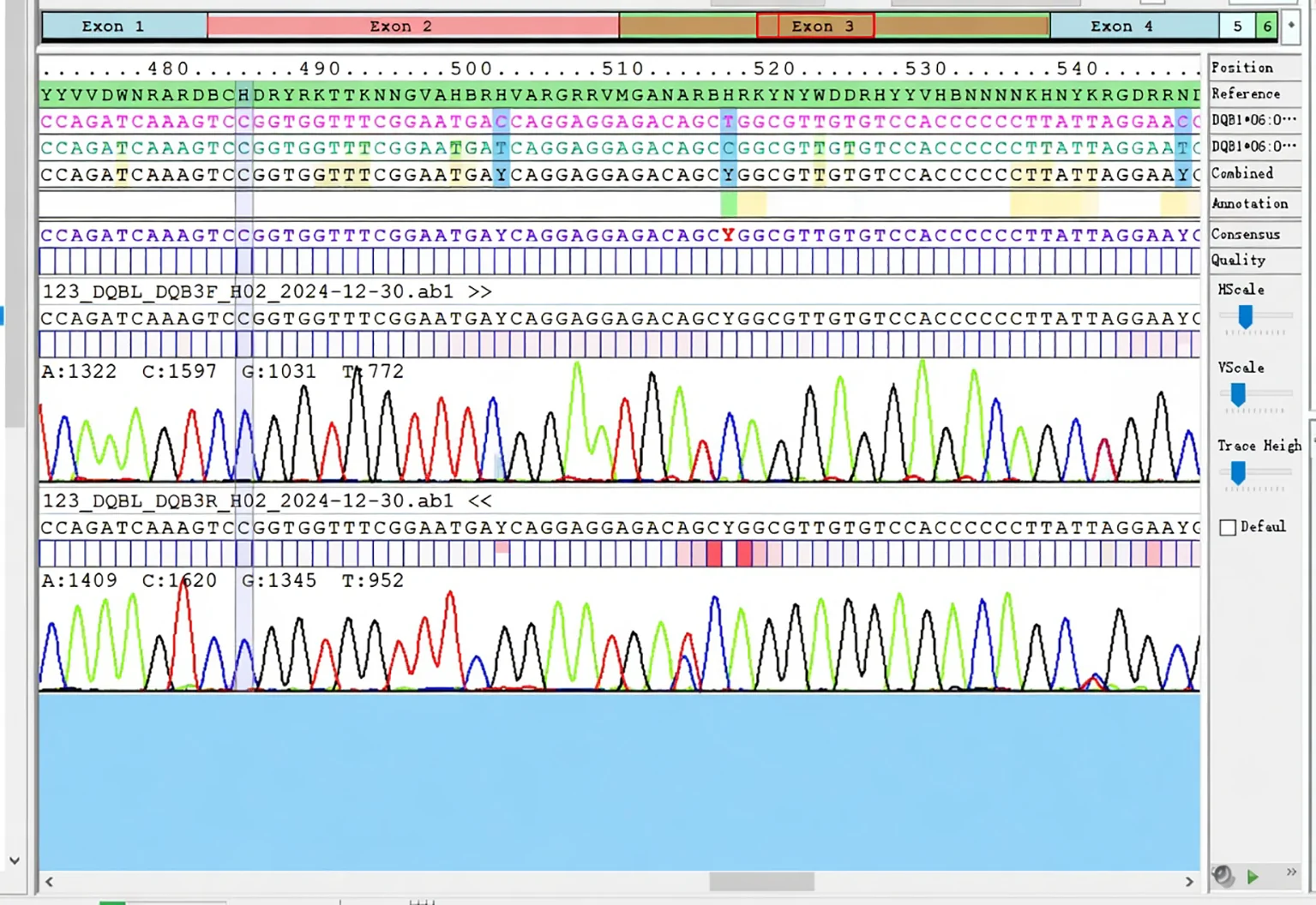
Genetic analysis of HLA DQB1 sequencing data.

The DNA sequence obtained from the raw data file was analyzed using HLA AccuTypeTM software, which generated the base sequence for the targeted gene site and compared it against its comprehensive HLA gene database. The chromatogram produced displayed peaks of varying colors, each representing specific nucleotide bases: green for adenine (A), blue for cytosine (C), Black for Guanine (G) and red for thymine (T). In the sequencing results for sample number 123 from the elderly participants, adenine (A), cytosine (C), and guanine (G) and thymine (T) were identified at various positions. Notably, this sample was randomly detected as DQB1*06:01 and DQB1*06:02 among the elderly individuals. These findings are crucial for determining the associated HLA-DQB1 alleles for this sample and are essential for genetic analysis and the identification of specific HLA-DQB1 alleles, as illustrated in **Figure 3**.

## 4 Discussion

In this study, different samples of elderly population were analyzed based on flow cytometry to detect RBC-bound antibodies,42 elderly individuals were found with IAG positive, indicating RBC-bound antibodies while in 41 individuals, the IAG was observed negative indicating no RBC-bound antibodies and both groups revealed a significant differences among various individuals. Our findings are an agreement with other studies; the impact of flow cytometry on Transfusion Medicine (TM) has been substantial, allowing for in-depth examination of individual cell populations and markers with sensitive, precise, and unbiased methods. Flow cytometry high sensitivity enables the detection of weak agglutination, a capability that traditional tube methods may lack. This makes flow cytometry a superior choice for identifying clinically significant antibodies compared to other techniques It serves as a dependable and highly sensitive tool that can potentially be explored as a novel standard diagnostic approach for Autoimmune Hemolytic Anemia (AIHA) (25–27).

The comparison of observed frequencies of elderly individuals with expected frequencies, as determined by the application of the Hardy-Weinberg law of equilibrium, yielded results shows a lack of significant deviation between the two. This finding suggests that the distribution of HLA alleles within our sampled population adheres closely to the theoretical expectations set forth by the Hardy-Weinberg equilibrium. The observed consistency in the distribution of these alleles among the elderly individuals studied provides valuable insights into the genetic makeup and diversity of the population under investigation. This alignment with Hardy-Weinberg expectations not only underscores the stability and equilibrium within the HLA allele frequencies but also lends support to the validity and robustness of our research study. The absence of significant variation in the distribution of these alleles further bolsters the reliability of our findings and reinforces the notion that the genetic composition of the population aligns with the fundamental principles of population genetics and inheritance. This consistency with the Hardy-Weinberg equilibrium serves as a foundational pillar for our study, affirming the accuracy of our results and enhancing the credibility of the conclusions drawn from our investigation.

The HLA, found on the short arm of human chromosome 6, displays multiple gene polymorphisms and boasts a complex structure. Its HLA class II genes, including DP, DQ, and DR sub regions, encode HLA class II molecules, facilitate antigen presentation, and play key roles in T cell activation, differentiation, and the adaptive immune response. Numerous studies have sought to clarify the link between DQB1 and autoimmune diseases (28, 29).

In this study, researchers employed flow cytometry to detect RBC-bound antibodies in a sample of elderly individuals. Subsequently, the genetic profiles of these participants were examined by analyzing their whole blood samples to identify various alleles associated with the index of agglutination and responsible for RBC-bound antibodies. Using the SBT technique, the research revealed a significant impact of the DQB1 *04 allele on IAG. In contrast, alleles such as *02, *03, *05, and *06 were found to have negligible effects on IAG. HLA allele DQB1 *04 suggests a possible association role in immune responses related to RBC-bound antibodies. By integrating these findings with a broader body of research, this study contributes to a comprehensive understanding of the complex interplay between HLA alleles, IAG, and immune responses in the elderly population.

Some other studies have reported that the likelihood of developing the first RBC alloantibody increases after the 40th blood transfusion (30). This study further supports our research, particularly in relation to the detection of RBC-bound antibodies in elderly individuals. The role of HLA polymorphisms in immune responses is significant, as evidenced by multiple studies linking specific HLA variations to conditions like ankylosing spondylitis, systemic juvenile idiopathic arthritis, systemic lupus erythematosus, and acquired idiopathic thrombotic thrombocytopenic purpura (31–35),these findings align with research demonstrating a consistent relationship between particular HLA-DQB1 alleles and the presence of antibodies targeting RBCs. Our finding of a potential association between HLA-DQB1*04 and RBC-bound antibodies in elderly individuals becomes more meaningful when viewed alongside previous studies. Research in transfusion-dependent thalassemia patients has demonstrated that HLA-DQB1*03 may have a protective role against alloantibody production (OR = 0.135, p=0.001), suggesting certain alleles can prevent alloimmunization (36). Similarly, in Chinese patient cohorts with combined auto- and alloimmunization, higher frequencies of HLA-DQB1*02 have been reported, indicating population-specific genetic susceptibilities (37).

Most genetic links identified to date have focused on chronically transfused recipients, such as those with sickle cell disease or thalassemia (38), or on patients with autoimmune conditions (39). Notably absent from the existing literature is any documented association between DQB1*04 and RBC-bound antibodies in aging populations experiencing immunosenescence. Recent studies in sickle cell disease have identified HLA Class II alleles that influence alloantibody development (overall estimate OR = 2.33) (38), while investigations in various populations have revealed genetic patterns associated with both alloimmunization and autoimmunization (40). Our findings complement this evidence by extending the investigation to a previously understudied population healthy elderly individuals experiencing age-related immune changes. The potential involvement of DQB1*04 in this context is particularly interesting given that this allele has not been prominently featured in studies of transfusion recipients or autoimmune patients. This raises the possibility that immunosenescence may involve distinct HLA-mediated mechanisms compared to those observed in chronic transfusion or autoimmune settings. Our observation that 88.9% of DQB1*04 carriers demonstrated RBC-bound antibodies compared to 48.4% of non-carriers suggests this allele may contribute to immune variability in aging.

Study Limitation: In our indirect flow cytometry method using serum with type O RBCs may detect a combination of true autoantibodies, alloantibodies, and naturally occurring antibodies, rather than exclusively measuring antibodies bound to the individual’s own RBCs *in vivo*. This limits our ability to definitively characterize these antibodies as autoimmune in nature. Direct antiglobulin testing (DAT) on patients’ own RBCs was not performed in this exploratory study, as samples were residual and not preserved for DAT; we strongly recommend including DAT in future studies to confirm *in vivo* antibody binding Additionally, clinical evidence of hemolysis (e.g., hemoglobin, bilirubin, LDH, reticulocyte count) was not assessed; therefore, the clinical significance of RBC-bound antibodies detected in this study remains unknown. Future studies should incorporate direct antiglobulin testing on patients’ own RBCs alongside indirect methods, include age-matched controls, and correlate laboratory findings with clinical markers of hemolysis to determine clinical relevance.

## 5 Conclusion

In conclusion, this exploratory study suggests a possible association between HLA-DQB1*04 and the presence of RBC-bound antibodies in elderly individuals, although this finding does not withstand correction for multiple comparisons and should be considered preliminary. The low frequency of this allele in our sample and the absence of a younger control group limit definitive conclusions about immunosenescence. Future studies with larger sample sizes are needed to validate these observations. Additionally, applying flow cytometry directly to patients’ own red blood cells may provide more specific information about *in vivo* antibody binding. Further investigation into the biological mechanisms underlying these associations, including correlation with clinical evidence of hemolysis, will help determine whether these laboratory findings have clinical significance. These initial observations provide direction for future research rather than definitive conclusions at this stage.

## Data Availability

The original contributions presented in the study are included in the article/supplementary material. Further inquiries can be directed to the corresponding author.
