# Correction: Detection of RBC-bound antibodies using flow cytometry and their correlation with HLA polymorphism

**DOI:** 10.3389/fimmu.2026.1950557

**Published:** 2026-10-19

**Authors:** Anwar Ullah, Lina Liu, Xia Qi, Hui Liu

**Affiliations:** College of Medical Laboratory, Dalian Medical University, Dalian, China

**Keywords:** agglutination, flow cytometry, HLA polymorphism, red blood cells, sequence-based typing

The **Author contributions** statement was erroneously given as: “AU: Writing – original draft, Writing – review & editing. LL: Conceptualization, Investigation, Methodology, Writing – original draft. XQ: Formal Analysis, Methodology, Resources, Writing –original draft. LH: Supervision, Writing – review & editing.”

The correct **Author contributions** statement is: “AU: Writing – original draft, Writing – review & editing. LL: Investigation, Methodology, Writing – original draft. XQ: Formal Analysis, Methodology, Resources, Writing –original draft. LH: Supervision, Writing – review & editing.”

The **Ethics statement** was erroneously given as: “The research followed the principles outlined in the Declaration of Helsinki. Approval for the study was obtained from the Dalian Medical University Ethics Committee, considering that the samples utilized were residual from clinical procedures and not collected specifically for this study, thereby posing no risk to the patients. The studies were conducted in accordance with the local legislation and institutional requirements. Written informed consent for participation in this study was provided by the participants’ legal guardians/next of kin.”

The correct **Ethics statement** is: “The research followed the principles outlined in the Declaration of Helsinki. Approval for the study was obtained from the Dalian Medical University Ethics Committee. Written informed consent was not required, as the samples utilized were residual from clinical procedures and not collected specifically for this study, were fully anonymized, and thereby posed no additional risk to the patients.”

The original version of this article has been updated.

